# Effect of the saliva biomolecules on the interface zirconia/Ti6Al4V triboactivity

**DOI:** 10.1080/07853890.2021.1897312

**Published:** 2021-09-28

**Authors:** H. Teixeira, A.P Serro, C. G. Figueiredo-Pina

**Affiliations:** a CDP2T and Department of Mechanical Engineering, School of Technology of Setúbal, Instituto Politécnico de Setúbal, Setúbal, Portugal; b Centro de Investigação Interdisciplinar Egas Moniz (CiiEM), Egas Moniz Cooperativa de Ensino Superior, Caparica, Portugal; c CQE, Instituto Superior Técnico, Universidade de Lisboa, Lisboa, Portugal; d CeFEMA, Instituto Superior Técnico, Universidade de Lisboa, Lisboa, Portugal

## Abstract

**Introduction:**

In some dental implants zirconia artificial dental crowns are fixed through a custom-built abutment made of a titanium alloy (Ti6Al4V) that, in turn, is tightly screwed to the implant. The rotational misfit at the implant-abutment hexagonal interface can lead to relative motion between the parts, resulting in interfacial wear. Usually, the wear tests are performed using artificial saliva (AS) without the addition of proteins. However, it is well documented that proteins can adsorb onto the biomaterials surface and may change the tribological response of the systems [1]. The objective of the present work was to evaluate the effect of theb main proteins of natural saliva on the tribological behaviour of the pair zirconia/Ti6Al4V.

**Materials and methods:**

Reciprocating pin on plate wear tests were performed using 6 mm balls of Ti6Al4V and plates of zirconia inside of a corrosion cell. The ball (working electrode) was connected to a potentiostat (GAMRY 600), through a wire and a calomel electrode was used as reference electrode. The wear tests were carried out in AS (composition described in [1]) without and with the addition of proteins (lysozyme 0.042 g/L + mucin 0.83 g/L + albumin 0.1 g/L) at 35 °C, during 2 h, with an applied load of 1 N, a sliding distance per cycle of 3 mm and a frequency of 1 Hz. The tests were performed after open circuit potential (OCP) stabilisation (Figure 1). OCP was recorded before, during and after wear testing.

**Results:**

The results showed that the presence of proteins leads to a reduction of the total wear and of the corrosion activity at the interface zirconia/Ti6Al4V during sliding, at the initial stage of the wear test. In fact, the wear rate decreases form ∼15 × 10^−5^ to ∼7 × 10^−5^ mm^3^/Nm. In addition, the OCP obtained during sliding for AS + proteins is much higher than the one observed for AS during the first 15 min and it equals the AS OCP just after 113 min of wear testing.

**Discussion and conclusions:**

The results suggest that the proteins adsorb at the sliding surfaces, forming a layer that acts as boundary lubricant. This layer prevents partially or totally the direct contact between the sliding surfaces and the total destruction of the passive film at the beginning of the tests (first 15 min). After the boundary film is disrupted, the passive film is totally destroyed, and the OCP drops to values near the ones observed for AS without proteins. In conclusion, the addition of proteins to saliva as an impact on the tribological response of the de sliding pair zirconia/Ti6Al4V.

**Figure 1.**
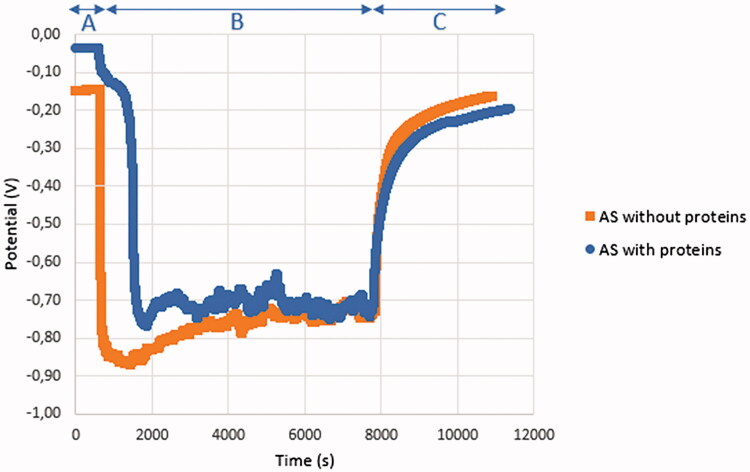
Open circuit potential recording during a) stabilisation (before wear testing), b) sliding and (wear testing) and C) recovery (after wear testing) in AS with and without proteins.
